# A brief review and clinical evidences of teriparatide therapy for atypical femoral fractures associated with long-term bisphosphonate treatment

**DOI:** 10.3389/fsurg.2022.1063170

**Published:** 2023-01-06

**Authors:** Jianpeng Gao, Xiao Liu, Xiaoyong Wu, Xiaoya Li, Jianheng Liu, Ming Li

**Affiliations:** ^1^ Department of Orthopaedics, Chinese PLA General Hospital, Beijing, China; ^2^ National Clinical Research Center for Orthopedics, Sports Medicine & Rehabilitation, Beijing, China

**Keywords:** teriparatide, atypical femoral fractures, bisphosphonate, subtrochanteric fracture, therapy

## Abstract

The risk of bisphosphonate (BP)-associated atypical femur fracture (AFF) has markedly increased over recent decades due to suppression of bone turnover, accumulation of structural micro-damage and reduction of bone remodeling consequent to long-term BP treatment. These medications further delay bone union and result in challenging clinical management. Teriparatide (TPTD), a synthetic human parathyroid hormone, exhibits unique anabolic effects and can increase bone remodeling and improve bone microarchitecture, further promoting fracture healing and reducing the rate of bone non-union. In this study, we briefly define AFF as well as the effects of BPs on AFFs, detailed the role of TPTD in AFF management and the latest clinical therapeutic findings. We have confirmed that TPTD positively promotes the healing of AFFs by reducing the time to bone union and likelihood of non-union. Thus, teriparatide therapy could be considered as an alternative treatment for AFFs, however, further research is required for the establishment of effective clinical guidelines of TPTD use in the management of AFF.

## Background

As the mainstay of osteoporosis treatment, bisphosphonates (BPs) can significantly decrease the risk of hip and vertebral fractures in osteoporosis in that these medications effectively increase bone mass and enhance bone strength (1, 2). However, an increasing number of studies have suggested that AFFs are closely associated with BP therapy due to consequent suppression of bone turnover, reduced remodeling and ultimately impaired healing capacity in the setting of long-term treatment (>5 years) (3–6). Despite a low incidence of AFFs (3.0–9.8 per 100,000 person-years) (7), such fractures are often resistant to therapy, result in poor bone union and high implant failure rates due to hardened bone and complete medullary canal obliteration (8–10). The mean healing time of AFFs postoperatively was 7.3 months (2–31 months) reported in literature; nearly a tenth of patients required revision surgery due to bone non-union or implant failure and approximately half of patients conservatively managed for incomplete fractures were eventually treated surgically (11). Furthermore, younger patients (aged <65 years) have tended to be more affected as compared to the older population (aged>65 years) (12). AFFs thus not only represent a serious health issue and enormous economic burden for patients, but also present a challenging clinical situation to manage for orthopedic surgeons.

Although the guidelines on the treatment of AFF have not been established, clinical trials have suggested that optimal AFF treatment includes surgical (intramedullary nailing and plate) and medical therapy. Medical management of AFF was summarized in the original task force report (American Society for Bone and Mineral Research, ASBMR) in 2010 and updated in the second task force report in 2014 (13, 14). In addition to cessation of BPs and supplementation of adequate calcium and vitamin D, a parathyroid hormone analogue termed teriparatide (TPTD) is likely to promote fracture healing. TPTD, the only US FDA-approved anabolic bone agent, has been reported by numerous studies to stimulate bone formation and remodeling, thus accelerating typical fracture healing (15, 16). The application of TPTD in the treatment of AFFs has also been reported. Several descriptive analyses have revealed TPTD could improve osteal micro-architecture and reduce bone non-union, exerting mechanisms of action opposite to BPs (17–19). Although a number of animal and clinical studies have reported beneficial effects of TPTD on AFFs associated with long-term BP therapy (20–23), lacking of integrated, effective treatment guidelines for the management of BP-associated AFF remains an orthopedic dilemma. Here, we briefly define AFF as well as detail the pathogenic mechanisms of AFFs in the setting of long-term BP therapy, the role of TPTD in AFF management and the latest therapeutic advances in order to provide more effective guidelines for successful clinical AFF management.

## Methods

**Figure 1:**
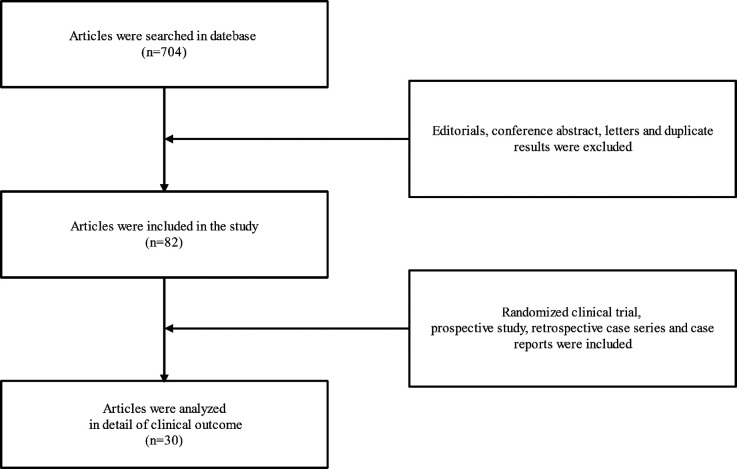
The flow chart of Information retrieval.

A search of PUBMED, EMBASE, and the Cochrane Central Register of Controlled Trials databases was conducted from 2001 to 2021. The search strategy was developed using MeSH terms and keywords associated with terms relevant to “Atypical femur fracture”, “Atypical femur fractures”, “femur fracture”, “femur fractures”, “Subtrochanteric fractures”, “Subtrochanteric fracture”, “Bisphosphonate”, “Bisphosphonates”, “BPS”, “BP”, “Teriparatide”, “Teriparatides”, “TPTD” and “TPTDS” in various combinations. A total of 704 articles were searched and the results were screened using the following inclusion and exclusion criteria. Inclusion criteria were available for all articles. Exclusion criteria included: (1) Duplicate results were excluded. (2) Editorials, conference abstract and letters were excluded. Two authors independently extracted information from the articles, which they read and reviewed the titles and abstracts and then the full text of the retrieved articles. 82 articles were deemed appropriate for our study. In addition, These included 3 articles from a prospective study, 7 articles from retrospective case series, and 20 case reports. These articles were then analyzed in detail of clinical outcome of TPTD therapy for AFFs (Figure 1).

## Results

### Definition of AFF

**Figure 2:**
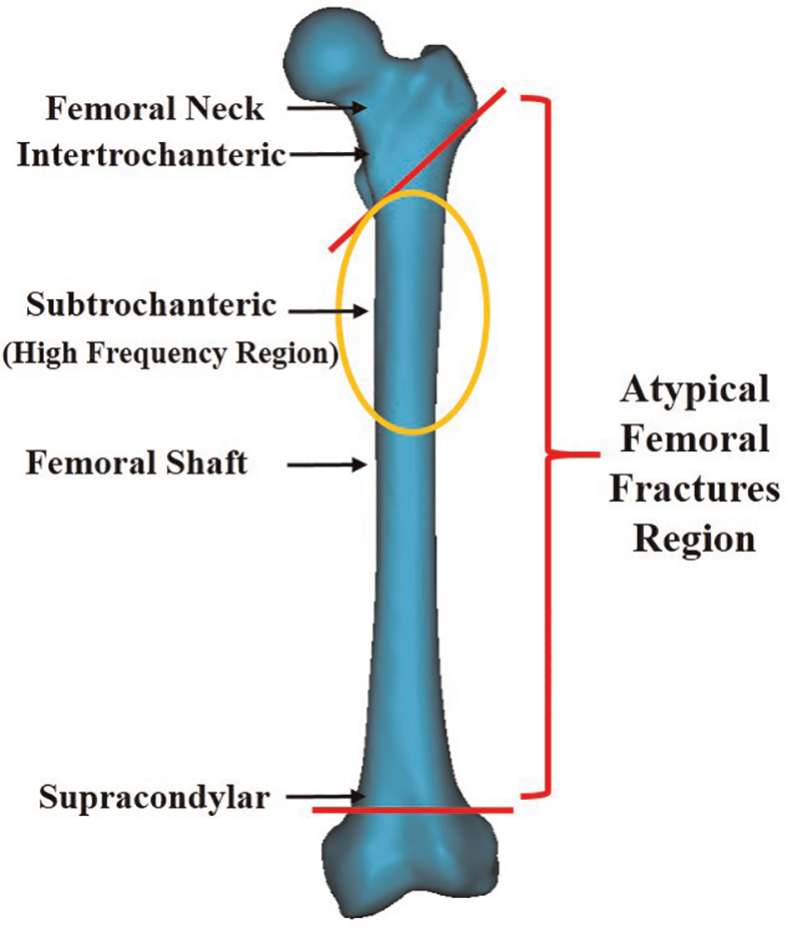
The region of atypical femur fractures, yellow circles denote areas of high incidence.

**Figure 3:**
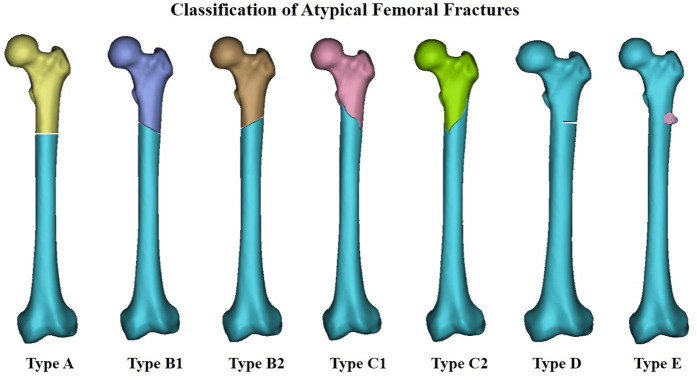
Classification of atypical femoral fractures; Type A implies transverse fracture, Type B implies short oblique fracture (which itself has2 subtypes), Type C implies spiral fracture associated with a medial spike (which itself has 2 subtypes), Type D implies incomplete fractures involving only the lateral cortex, Type E implies localized periosteal reaction in the lateral cortex.

Since the publication of the article suggesting an association between BPs and AFFs in 2005 (24), a growing number of case reports and series have revealed a high correlation between BP therapy and AFFs. Fractures were reported to be primarily located along the femoral diaphysis from just distal to the lesser trochanter to just proximal to the supracondylar flare (Figure 2) (13). These fractures were characterized as spontaneous and resulting from low-energy injury. They were usually not comminuted and fractured in the subtrochanteric area, or the proximal one-third of the femoral shaft. In order to clearly define AFF, the American Society for Bone and Mineral Research (ASBMR) established 5 major and 7 minor features of AFFs in the original 2010 task force report. All major features are required to satisfy the case definition of an AFF; none of the minor features are required, but occasionally some have been associated with such fractures. The 5 major features included: (1) fracture location anywhere along the femur from just distal to the lesser trochanter to just proximal to the supracondylar flare; (2) lack of association with trauma, or association with minimal trauma, as in a fall from a standing height or less; (3) transverse or short oblique fracture configuration; (4) non-comminuted nature; (5) fracture extension through both cortices and association with a medial spike; incomplete fractures involve only the lateral cortex. The 7 minor features included: (1) Localized periosteal reaction of the lateral cortex, often referred to in literature as beaking or flaring; (2) generalized cortical thickness increase of the diaphysis; (3) presence of prodromal symptoms such as dull or aching pain in the groin or thigh; (4) bilateral fractures and symptoms; (5) delayed healing; (6) comorbid conditions (e.g., vitamin D deficiency, RA, hypophosphatasia); (7) use of pharmaceutical agents (e.g., BPs, glucocorticoids, proton pump inhibitors). In order to standardize and improve diagnostic criteria, the definition of AFFs was revised in the second 2014 task force report. The major alterations were the establishment of clearly defined transverse or short oblique AFFs configurations, inclusion of minimal comminuted fractures, and the changing of the periosteal or endosteal lateral cortex stress reaction at the fracture site from minor to major categories(13, 14). Based on it, we classified AFFs into 5 types (Figure 3) according to their characteristics, providing further clarification.

### BP influences on AFFs

BP, consisting of P-C-P bonds, not only imbues strong affinity for hydroxyapatite (HAP) binding and in turn prevents both HAP crystal growth and dissolution, but also directly or indirectly inhibits osteoclast-mediated resorption and remodeling of bone (25–28). Via these pathways, BP can prevent bone loss, reduce bone turnover and increase overall mineralization (29), and widely used in the management of osteoprosis, pagets disease of the bone and osteocarcinomas. Throughout treatment, BPs are incorporated into newly formed bone and persist there for long time. In addition, a continuous decrease in chronic bone turnover likely leads to greater bone fragility (30). The research had proved that the risk of atypical femur fracture increased with longer duration of BP use and rapidly decreased after BP discontinuation. In addition, Asians had a higher risk than Whites.

Table 1 details how BP treatment predisposes to AFFs development. Due to the high affinity of BP to HAP binding and long-term effects on bone resorption, a marked decrease in bone remodeling delays prompt micro-fracture healing (31). As micro-fractures accumulate, a greater stress reaction results. This is the reason x-ray imaging reveals these fractures to exhibit focal lateral cortical thickening, or transverse, short oblique fracture lines as the stress reaction develops. Moreover, BPs decrease both crystal formation and dissolution, thereby narrowing crystal distribution without affecting mean crystal size and increasing overall mineralization (32). As bone turnover is reduced, BPs prevent maturation of collagen and increase levels of advanced glycation end products (33). Due to alteration in the properties of the HAP and bone matrix, bone tissue homogenizes and is less able to reduce local stress. This enhances energy distribution that makes the bone more brittle and predisposes to fracture (34). Studies have confirmed BP to directly suppress vasculogenesis and retard the remodeling of calcified cartilage calluses into mature bone during fracture healing (35, 36). AFFs healing is thus typically prolonged and may even result in bone non-union. Appropriate treatment strategies are essential for successful AFFs management, and TPTD, with its unique bone-forming effects, is likely a good choice.

**Table 1:** Influences of BPs on AFF and the role of TPTD in AFF management.

Atypical Femoral Fractures: Likely Pathogenesis and Therapeutic Strategy
**BPs predispose to AFF**
• Chronically decrease bone remodeling • Results in accumulation of minor damage and fractures • Alter the distribution and size of hydroxyapatite crystals and result in excessive mineralization • Inhibit collagen maturation and increase glycosylated product content • Reduce bone heterogeneity • Directly inhibit angiogenesis and delay callus calcification
**The Role of Teriparatide in AFF**
• Promotes remobilization and redistribution of BPs within bone tissue • Decrease BP content within calluses during fracture healing • Replaces fully mineralized bone matrix with newly synthesized and less completely mineralized matrix • Decreases glycated collagen production and increases expression of new type II and X collagen • Increases bone heterogeneity • Anabolism promotes bone formation and decreases adipogenesis in cancellous bone

### The role of teriparatide in AFFs

TPTD has mainly been used to treat osteoporosis in postmenopausal women and older men since 2002 after its approval by the FDA for this purpose (37). It is manufactured using a genetically modified strain of E. coli and consists of a 1–34 N-terminal amino acid sequence of the intact human parathyroid hormone while maintaining whole parathyroid hormone (PTH) biological activities (38). The current recommended dose is 20 µg once daily by subcutaneous injection in a sustained treatment cycle for no greater than 2 years. Numerous studies have reported TPTD to directly activate osteoblast *via* binding to their PTH receptors, thus increasing osteoblast genesis and reducing osteoblast apoptosis (39). TPTD increases production of growth factors and cytokines, such as IGF-1 and TGF-β, resulting in pre-osteoblast recruitment from marrow stromal cells (40, 41). TPTD also positively affects cell-signaling pathways, such as IHH, Wnt/β-catenin and AC/cAMP-PKA and promotes osteoblast proliferation and differentiation (42–44). TPTD was furthermore confirmed to reduce sclerostin, an inhibitor of bone formation in both rodents and humans (45, 46). TPTD thus possesses significant advantages in the promotion of bone formation and is considered a good choice for the treatment of fractures (47, 48).

Fractures are understood to undergo primary and secondary healing. Primary healing or direct bone healing is characterized by fractured bone cortex ends directly uniting and results in restoration of anatomy and rigid internal fixation. Secondary healing, or indirect bone healing, is similar to embryonic bone formation and includes intramembranous and endochondral ossification *via* callus formation. Fractures typically heal *via* secondary healing which the processes involved are indeed complex (49). A better physiological environment decreases chances of delayed union or non-union occurring. In the setting of osteoporosis, poor osteoblast function and inappropriate callus maturity, coupled with the effects of BPs, result in frequent complications of bone injury. However, TPTD, exerting anabolic effects as opposed to BP (Table 1) has been confirmed to significantly redistribute BPs within fracture sites in some studies, even reduce BP content with prolonged use of this drug (50). Moreover, because of anabolism leading to increased bone turnover and remodeling, TPTD not only replaces fully mineralized bone matrix with newly synthesized and less completely mineralized matrix but also decreases production of glycated collagen while increasing expression of new type II and X collagen (51, 52). Such alterations in mineralized bone and matrix result in preferable osseous heterogeneity. Furthermore, fracture callus size, density and volume become significantly increased (51). Interestingly, intermittent TPTD administration was found to promote osteoblast genesis and decrease adipogenesis at the site of cancellous bone, resulting in better bone union (53). Dobnig et al. (54) found that changes in iliac crest microdamage in previously treatment-naive patients managed with TPTD, or in patients switched from alendronate to TPTD, entailed decreased crack density (Cr.Dn), crack surface density (Cr.S.Dn) and crack length (Cr.Le) when compared to patients previously treated with alendronate after 24 months of TPTD administration. These findings demonstrate that TPTD reduces accumulation of bone microdamage in postmenopausal women previously treated with alendronate. Similarly, Paul et al. (21) studied 15 patients referred to The Colorado Center for Bone Research (CCBR) after they suffered BP-associated AFF using quantitative iliac crest histomorphometry both before and after 12 months of TPTD (20 µg SQ/day). Results revealed that discontinuation of BPs and administration of TPTD was associated with improvement in bone turnover and an increase in all 3 dynamic histomorphometric parameters; including bone formation, mineralized surface, and mineral apposition. Although a number of studies have verified the beneficial effects of TPTD on AFF healing, the mechanism of TPTD action in the setting of AFFs remains unknown. Based on prior research, TPTD has unique potential in the treatment of AFFs. Also a large number of clinical evidence has additionally found that TPTD therapy can be considered as an alternative treatment for AFF.

### Clinical outcome of TPTD therapy for AFFs

The management of BP-associated AFFs varies due to AFF type (i.e., stress reaction; stress fracture; incomplete or complete subtrochanteric or femoral shaft fracture). On account of high rates of poor union and surgical difficulties, neither conservative nor operative management are capable of achieving good treatment outcomes. TPTD supplementation, however, reducing the time to bone union and likelihood of non-union, positively affects AFF healing (55, 56). Several case reports as well as retrospective (Table 2) and prospective studies (Table 3) have demonstrated the efficacy of TPTD in the treatment of AFFs (17–19, 21, 57–82).

**Table 2:** Summary of the retrospective studies of TPTD therapy for AFF.

First author/reference/date/country	Patients (n)	TPTD therapy (n)	Age/mean age	Gender	BP duration/mean time	Fractures (n)	Location	Initiation of TPTD	Treatment dose	Duration of treatment	surgery (n)	Union rate (%)	Time to union/mean time
Anas Saleh, 2012, USA (57)	10	9	50–85 years/67 years	F	4–17 years/10 years	IF	ST (4)/FD (11)	NS	NS	24 months	NS	50	3 months
Paul D. Miller, 2015, USA (21)	15	13	57–87 years/67 years	F	6–11 years/7 years	CF	B, FD (*n* = 6)/l or R FD (*n* = 9)	After the first bone biopsy	20 µg/d	12 months	IN	NS	NS
Naohisa Miyakoshi, 2015, Japan (58)	34	NS	66–88 years	F	1–11.6 years/5 years	NS	NS	NA	20 µg/d, 56.5 µg/w	NS	IN (33)/P (4)	93.7	2–7 months/5.4 months
Basmah K. Alwahhabi, 2017, Saudi Arabia (59)	34	23	46–89 years	F (*n* = 33)/M (*n* = 1)	4 years and more	NS	FD (*n* = 16)/TD (*n* = 4)/PF (*n* = 1)	NS	20 µg/d	18–24 months (*n* = 16)	IN/P	52.4	NS
Wen-Ling Yeh, 2017, Taiwan (60)	13	NS	58–79 years/70.2 years	F	2.5–6 years	CF	ST (10)/FD (6)	Postoperation immediately	20 µg/d	NS	IN	75%	2.3–8.2 months/4.4 months
H. Tsuchie, 2018, Japan (61)	43	43	57–88 years/78.8 years	F (42)/M (1)	1–12 years/5.2 years	CF (*n* = 30)/IF (15)	ST (*n* = 3)/FD 42	NS	20 µg/d (*n* = 32), 56.5 µg/w (*n* = 13)	NS	IN (35)/P (7)	100%	6.1 ± 4.1 months (daily)/10.1 ± 4.2 months (weekly)
HiroyukiTsuchie, 2021, Japan (62)	113	99	79.2 years	F	NS	IF (1)/CF (12)	NS	Postoperation immediately (*n* = 7)/6 months (*n* = 6)	20 µg/d	12 months	NS	NS	NS

AFF, atypical femur fracture; AR, antiresorptive; NS, not stated; TPTD, teriparatide; F, female; M, male; ST, subtrochanteric; FD, femoral diaphysis; PF, pelvic fracture; CF, complete fracture; IF, incomplete fracture; IN, intramedullary nail; P, plate; IM, immediately; B, bilateral; R, right; F, left.

**Table 3:** Summary of Prospective Studies of TPTD Therapy for AFF

First author/reference/date/country	Patients (n)	TPTD therapy (n)	Age/mean age	Gender	BP duration/mean time	Fractures (n)	Location	Initiation of TPTD	Treatment dose	Duration of treatment	surgery (n)	Union rate (%)	Time to union/mean time
Cherie Ying Chiang, 2013, Australia (63)	14	5	76 years	F (13)/M (1)	4–10 years	IF (8)/CF (6)	NS	NS	20 µg/d	6 months	NS	NS	NS
Nelson B Watts, 2017, USA (64)	14	14	52–83 years/68.3 years	F	3–14.5 years/8.8 years	IF (5)/CF (9)	NS	NS	NS	24 months	NS	64.3	NS
S. L. Greenspan, 2017, USA (65)	13	13	74.2 years	F	NS	IF (1)/CF (12)	NS	Postoperation immediately (*n* = 7)/6 months (*n* = 6)	20 µg/d	12 months	NS	NS	NS

AFF, atypical femur fracture; AR, antiresorptive; NS, not stated; TPTD, teriparatide; F, female; M, male; SF, subtrochanteric; FD, femoral diaphysis; PF, pelvic fracture; CF, complete fracture; IF, incomplete fracture; IN, intramedullary nail; P, plate; IM, immediately; B, bilateral; R, right; F, left.

Gomberg et al. (68) initially reported TPTD use in AFF management. The patient was a postmenopausal woman treated with a BP for 13 years. Obvious reduction of edema, pain and appearance of faint cortical bridging at fracture sites after 6 months of continuous TPTD treatment was noted. Then, a number of case reports or series subsequently found TPTD use for AFF treatments could reduce periosteal reaction and promote microdamage repair in the setting of conservative or surgical treatment. In addition to pain reduction and minimization of dysfunction, alkaline phosphatase levels in AFF patients who suffered hypophosphatasia were effectively restored with TPTD administration (74). Treatment of TPTD is usually started for diagnosis or after surgery. The dose is usually 20 µg/d and the treatment cycle ranges from 3 to 24 months. Calcium and vitamin D were often used in combination. It is worth emphasizing that TPTD combined with stable fixation results in satisfactory AFF outcomes in most cases. Almost all patients had clinical outcomes of bone healing, and the healing time was between 1 and 6 months.

Saleh et al. (57) first retrospectively investigated 10 patients that suffered a total of 14 incomplete AFFs. 5 of 14 fractures did not manifest with a radiolucent fracture line and were healed conservatively with TPTD. Radiolucent fracture lines across thickened cortices were noted in 9 fractures. Two of these fractures responded to 3 months of conservative therapy with TPTD and were found to have completely healed on radiography. Six of these fractures chose surgical prophylaxis after 3 months of conservative management, whereas 1 patient underwent surgical prophylaxis without a trial of conservative management. Miyakoshi et al. (58) retrospectively reviewed the medical records of 45 consecutive AFFs in 34 Japanese patients who had been treated with oral BPs for osteoporosis prior to their AFFs and followed for a period of 12 months. A total of 37 complete or incomplete AFFs were treated surgically and 8 incomplete AFFs were treated conservatively. Patients were non-randomly divided into non-TPTD (*n* = 24) and TPTD (*n* = 21) groups. Results revealed the healing time of all surgically-treated AFFs to have been significantly shorter in the TPTD group and the frequency of delayed healing or non-union to have been significantly lower in the TPTD group as well. Although similar findings were noted in surgically treated complete AFFs, sub-analyses of incomplete AFFs treated conservatively revealed no significant differences between groups. Hence, TPTD was found to markedly accelerate fracture healing postoperatively that would otherwise have been delayed. Chiang et al.'s (63) prospective study involved 14 patients that consecutively presented with AFFs over a course of 2 years. Of these, 5 were offered TPTD therapy while the remaining 9 were not due to contraindications. The 5 patients who had undergone TPTD therapy were found to have a 2–3 folds increase in bone remodeling markers and more rapid fracture healing. Of the 9 patients managed conservatively or surgically, 7 suffered poor fracture healing with ongoing pain, 1 sustained a contralateral atypical fracture and 1 experienced fracture union only after 1 year. Similar results were reported in Yeh's (60) study, 13 patients who suffered AFFs were all treated with intramedullary fixation, 6 patients underwent TPTD treatment initially for at least 6 months while others did not. The mean time to bone union was 4.4 months in the TPTD treatment group and 6.2 months in the non-TPTD treatment group. The means of the modified Harris Hip Score and Numerical Rating Scale were significantly better in the TPTD group at 6 months postoperatively. TPTD was thus confirmed to greatly assist in fracture healing, hip function recovery, and pain relief. A randomized pilot clinical trial confirmed immediate therapy with TPTD to be superior for fracture healing in the setting of AFFs compared to a 6-month delay in TPTD therapy and effectively prevented a decrease in bone density (65).

Although TPTD was found to initially promote rapid healing of AFF, the development of new, contralateral AFFs highlights that TPTD does not indefinitely protect against future AFF occurrence. TPTD may thus be limited in its ability to reverse skeletal side effects of long term BP therapy. Anti-resorptive therapies have also been implicated in the development of contralateral AFFs in the setting of adjunctive strontium ranelate therapy (77). Moreover, Watts et al. (64) conducted a prospective and open-label study involving 14 AFF patients who were previously treated with BPs, examining changes in bone mineral density, trabecular bone score, bone turnover markers, and fracture healing, as well as quantitative histomorphometry, after a 24-month TPTD treatment period. At 24 months, 6 patients were found to have healed fractures, 3 to have partially healed fractures, 2 with unchanged fractures and 1 with nonunion. However, in a patient who suffered 2 successive fractures, the fracture that occurred prior to TPTD treatment was reported as healed while the fracture that occurred during TPTD treatment was found to be only partially healed. No significant effects of TPTD on hip bone mineral density, mineralizing surface to bone surface ratio, or trabecular bone score were noted. In addition, no consistent effect on fracture healing was noted, implying that sole use of TPTD is likely not reliable for rapid healing of AFFs. TPTD should, however, be understood as exerting beneficial effects on AFF healing. A greater amount of high level of evidence, in particular randomized controlled trials, should be performed to further confirm the efficacy of TPTD in AFF management.

## Discussion

Long-term application of BPs, affecting bone mass and resulting in AFF, made the treatment of AFFs difficult. Due to its unique biological effects, TPTD has been demonstrated in basic experiments to reverse the effect of BP on bone and promote the healing of AFFs, and has achieved good results in clinical application. Based on the articles we reviewed, it is not difficult to find that TPTD has certain advantages in promoting the healing of complete or incomplete AFFs, especially in combination with strong internal fixation and medical supplement with calcium, cholecalciferol and vitamin D.

Based on the existing clinical evidences, especially the prospective and RCT studies, it provides certain clinical basis for the TPTD treatment of AFFs. Hence, we have developed a preliminary diagnosis and treatment process based on the latest clinical findings to assist AFF treatment. Firstly, regular imaging examinations (MRI is recommended) are recommended for patients who have been using BPs for a long time (especially those who have been using BPs for more than 5 years), regardless of whether they have symptoms of thigh pain, for the purpose of early detection and treatment. Secondly, BPs therapy should be stopped immediately after AFF diagnosis, and conservative or surgical treatment should be given according to fracture type. Thirdly, it is recommended to apply TPTD (20 µg/day, 12–24 months) immediately after diagnosis for patients receiving conservative treatment and patients receiving surgery, and calcium, cholecalciferol and vitamin D should be combined. Fourthly, surgical intervention is recommended as soon as possible for patients with ineffective conservative treatment, and preventive surgical treatment is also recommended for patients with initial symptoms or incomplete fractures. Fifth, regular monitoring of blood calcium after the application of TPTD is recommended, and the treatment cycle depends on the time of fracture healing condition, and it is recommended that the application should not exceed 24 months. Replacement of other drugs such as Denosumab is recommended after the cessation of TPTD treatment. We hope that AFFs can be easily treated through the above treatment process.

But so far, even though AFF has been studied a lot, it still has some shortcomings. In general, the mechanism by which TPTD promotes AFF healing is unclear. In addition, we found that most of the current clinical studies were case reports and retrospective studies with low level of evidence. The specific time of TPTD initiation, dose and cycle of application, and the difference between conservative and surgical treatment were not clear. Therefore, more high-level clinical studies are needed to confirm the efficacy of TPTD in treating AFFs.

## Conclusion

BP-associated AFFs present numerous difficulties to both clinicians and patients. An effective treatment is thus urgently needed. Basic and clinical research has revealed TPTD to exert a unique anabolic effect in AFF healing and underscores its importance as an alternative treatment for this condition. Further research, however, is required for the establishment of effective clinical guidelines of TPTD use in the management of AFFs.
